# Electrochemical biosensors on microfluidic chips as promising tools to study microbial biofilms: a review

**DOI:** 10.3389/fcimb.2024.1419570

**Published:** 2024-09-24

**Authors:** Adei Abouhagger, Raimonda Celiešiūtė-Germanienė, Neringa Bakute, Arunas Stirke, Wanessa C. M. A. Melo

**Affiliations:** Department of Functional Materials and Electronics, State Research Institute Centre for Physical Sciences and Technology (FTMC), Vilnius, Lithuania

**Keywords:** microbial biofilms, electrochemical biosensors, microfluidics, real-time monitoring, biofilm dynamics

## Abstract

Microbial biofilms play a pivotal role in microbial infections and antibiotic resistance due to their unique properties, driving the urgent need for advanced methodologies to study their behavior comprehensively across varied environmental contexts. While electrochemical biosensors have demonstrated success in understanding the dynamics of biofilms, scientists are now synergistically merging these biosensors with microfluidic technology. This combined approach offers heightened precision, sensitivity, and real-time monitoring capabilities, promising a more comprehensive understanding of biofilm behavior and its implications. Our review delves into recent advancements in electrochemical biosensors on microfluidic chips, specifically tailored for investigating biofilm dynamics, virulence, and properties. Through a critical examination of these advantages, properties and applications of these devices, the review highlights the transformative potential of this technology in advancing our understanding of microbial biofilms in different settings.

## 1 Introduction

Microbial biofilms are complex and dynamic adhesive communities of microorganisms that are enclosed by a matrix of extracellular polymeric substances (EPS), resulting in the clustering of microbial cells (Hall-Stoodley et al., 2004; Yin et al., 2019). Bacterial adhesion to biological or non-living surfaces initiates the biofilm formation process. During this process, bacteria adhere to the surface and subsequently produce the EPS matrix, marking the initiation of biofilm development (Yin et al., 2019). The resulting biofilm structure provides a higher chance of survivability for microbial cells by providing a protective microenvironment against different potential environmental challenges including the host immune system, antibiotics, dehydration, and salinity (Santos et al., 2018; Yin et al., 2019). Consequently, microbial biofilms account for more than 75% of hospital-acquired infections and are a common contributor to chronic persistent infections (Costerton et al., 1999; Sharma et al., 2019). This includes chronic wound infections (Evelhoch, 2020), implant-related infections (Stewart and Bjarnsholt, 2020) and chronic urinary tract infections (UTIs) (Trautner and Darouiche, 2004). The urgent nature of these clinical concerns is heightened due to the lack of clearly established antibiofilm treatments and straightforward methods for growing and investigating biofilms in laboratory settings. Recognizing the importance of analyzing biofilms is crucial for the development of new treatments aimed at addressing these pressing medical issues.

In this context, the need to create new biofilm research techniques is becoming more pressing due to the continuous difficulties in comprehending and eliminating microbial biofilms. Therefore, electrochemical biosensors have emerged as promising tools for monitoring biofilm dynamics. Their real-time capabilities, non-destructive nature (Ameer et al., 2023), and ability to measure various biofilm parameters, including metabolic activity, virulence factors, and changes in pH (Saccomano et al., 2021), positioned them as accessible and comprehensive tools for studying biofilm behavior.

Considering the dynamic nature inherent to microbial biofilms, marked by intricate interactions and adaptive behaviors, there arises a need for methodologies that can both monitor biofilm dynamics and provide a controlled environment for their growth. While electrochemical biosensors excel in real-time insights, the integration of microfluidic systems takes our exploration a step further. Microfluidics with its precision and ability in manipulating small fluid volumes emerges as an ideal companion to electrochemical biosensors offering a regulated and dynamic microenvironment for studying biofilm behavior (Guliy et al., 2022). This integration enables the study and analysis of biofilm dynamics within a regulated flow of nutrients and shear force, providing dynamic platforms for *in vitro* biofilm monitoring.

**Figure 1:**
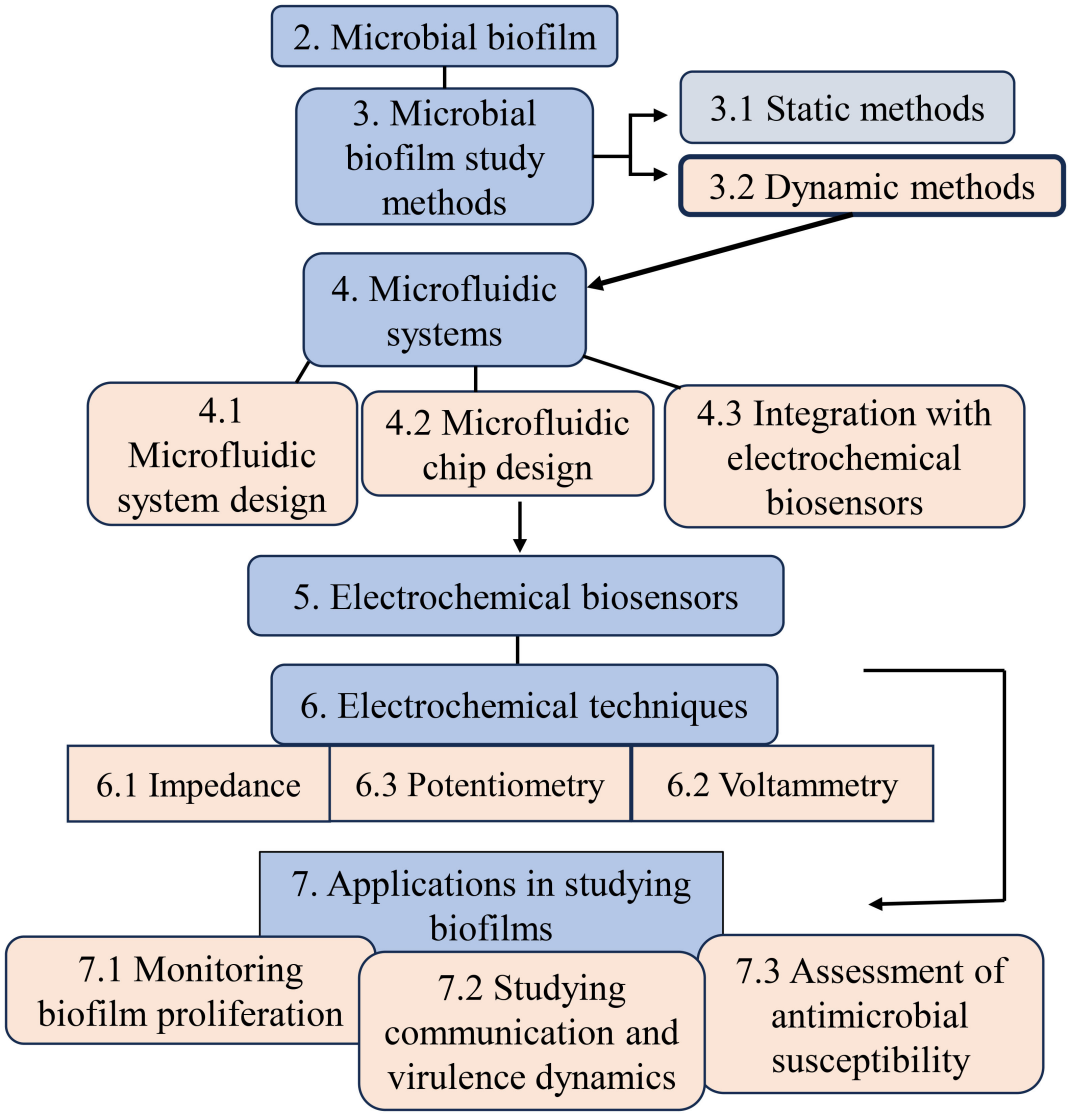
An overview flow chart of the review topics.

In this review, we dive into the foundational principles of electrochemical biosensors and their transformative integration into microfluidic chips. Subsequently, we will cover a variety of aspects of electrochemical microsystems designed for the study of microbial biofilms, including the development of biosensors, the integration of microfluidic chips, and electrochemical techniques (**Figure 1**). By exploring these elements, we clarify different aspects of studying biofilm behavior, covering growth dynamics, virulence factors, and the evaluation of antibiofilm agents. This review provides an insight into the current findings and potential future directions in electrochemical dynamic microfluidic systems for biofilm research.

## 2 Microbial biofilms

Microbial biofilms can be beneficial or malefic in nature with their prevalence on Earth, ranging from 40% to 80%, have prompted extensive research into their multifaceted nature (Flemming and Wuertz, 2019). While historically regarded for their negative impacts, it’s increasingly recognized that bacterial biofilms can also yield beneficial effects (Rosche et al., 2009). This understanding has spurred interest in leveraging biofilms for agricultural and industrial applications, including biological control against phytopathogens, bioremediation of pollutants, wastewater treatment, marine ecosystem protection, and corrosion prevention (Muhammad et al., 2020).

Conversely, biofilm formation can pose significant challenges, particularly in settings such as food processing facilities and hospitals (Coughlan et al., 2016). Pathogenic biofilms in these environments contribute to food spoilage, endanger consumer health, and perpetuate persistent infections on medical devices and tissues (Galié et al., 2018). The detrimental impact of biofilms extends beyond immediate health concerns, with implications for antibiotic resistance and the persistence of microbial infections (National Institutes of Health, NIH). Reports from the Centers for Disease Control highlight the staggering toll of biofilm-associated infections, both in terms of human lives and economic burden (Centers for Disease Control, 2007).

Biofilms exhibit diverse pathological characteristics and are ubiquitous, found in numerous environments including medical implants, living tissues, water channels, pipes, hospital facilities, food processing sites, and various other biotic and abiotic surfaces (Donlan, 2002; Donelli and Vuotto, 2014). Biofilm-associated microorganisms undergo changes in phenotype and gene expression, leading to resistance against established antibiotics, decreased metabolic activity and growth rates, and heightened production of virulence factors (Sharma et al., 2015). According to data from the NIH, microbial biofilms are responsible for approximately 65% of microbial infections and up to 80% of chronic infections, affecting both tissue integrity and implanted medical devices.

Centers for Disease Control reported from 2007, there were approximately 1.7 million instances of hospital-acquired infections, leading to over 0.5 million deaths, and imposing an economic burden exceeding US$11 billion for treating biofilm-associated infections (Brinkman et al., 2016). Moreover, various sectors of the food industry, including poultry, dairy, ready-to-eat foods, and aquaculture, suffer significantly from biofilm-producing microorganisms, resulting in food spoilage, disease outbreaks, and fatalities (Giaouris and Simões, 2018). Consequently, given the widespread prevalence of biofilm-associated microorganisms and the limited efficacy of current antibiotics, there is a compelling need to transition towards the development of non-toxic and potent antibiofilm agents. These agents should target signaling pathways that regulate quorum sensing (QS), EPS synthesis, biofilm-related genes, microbial motility, adhesion, dispersion, and other critical factors (Li and Lee, 2017; Parrino et al., 2019; Rather et al., 2021b).

**Figure 2:**
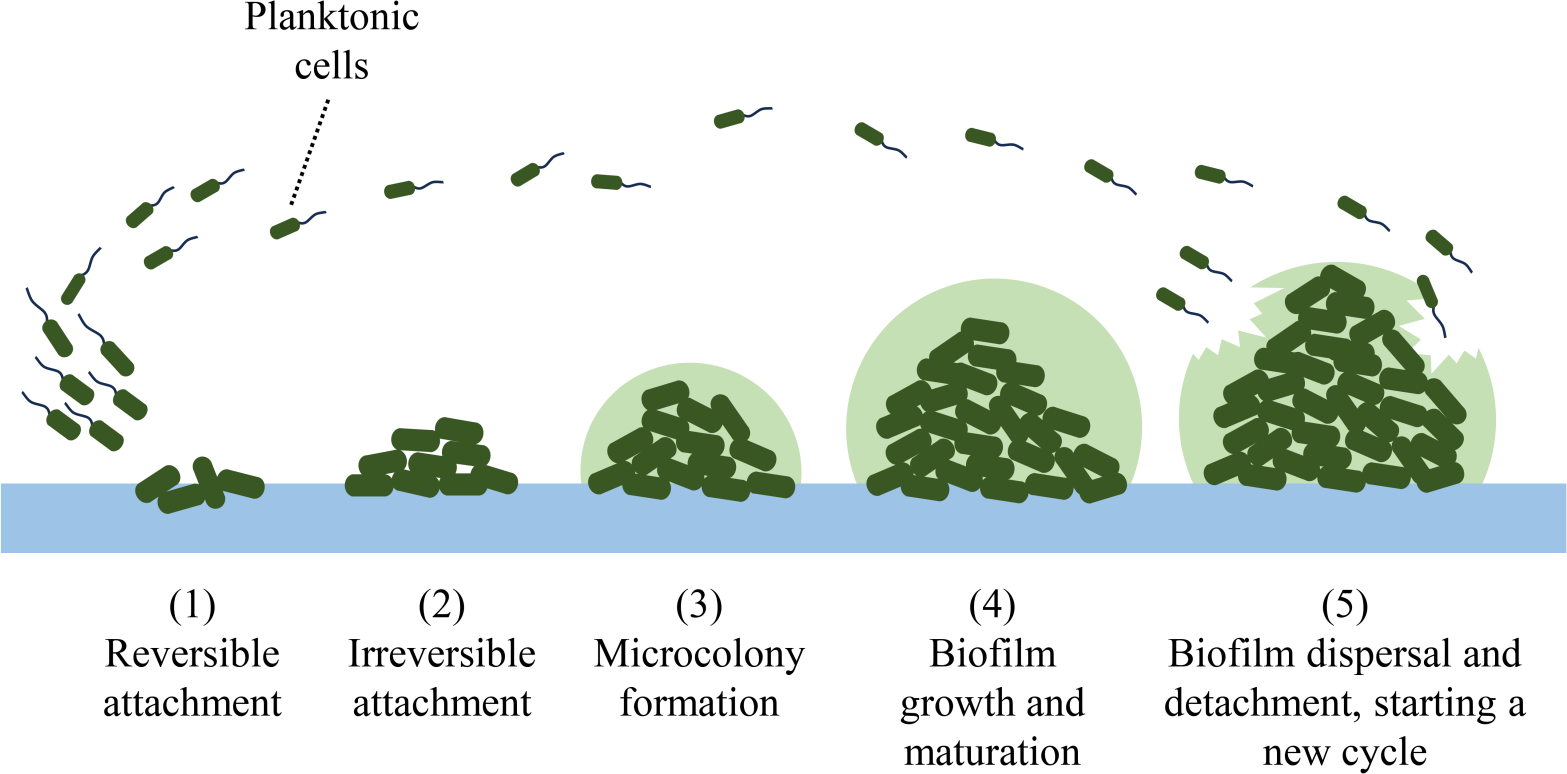
Biofilm formation stages.

The process of biofilm formation unfolds through a series of distinct stages. Firstly, in stage 1) reversible attachment, cells attach polarly to surfaces, allowing for easy detachment. Moving to stage 2) irreversible attachment, cells flatten against surfaces, becoming firmly entrenched through electrostatic forces, and resisting displacement by environmental factors. Stage 3) microcolony formation ensues, marked by the accumulation of microbes and the secretion of extracellular polymers. Stage 4) growth and maturation witness the emergence of a three-dimensional (3D) biofilm structure, accompanied by the production of QS molecules and surfactants. Finally, in stage 5) dispersion, microbial biofilms detach in response to disruptive factors like catabolite repression, nutrient scarcity, and secretory proteins (Rather et al., 2021a). These sequential stages of biofilm formation are illustrated in **Figure 2**.

Understanding biofilm behavior is crucial for various fields, including medicine, industry, and environmental science. Thus, having devices and methodologies that can accurately assess biofilm behavior is imperative for developing effective strategies to control harmful biofilms, optimize industrial processes, and harness the beneficial aspects of biofilm communities. These devices enable researchers to study biofilm formation, growth, and dispersal under controlled laboratory conditions, providing valuable insights into the underlying mechanisms and dynamics of biofilm development (Azeredo et al., 2017). Furthermore, advanced imaging and sensing technologies allow for real-time monitoring of biofilm behavior, facilitating the observation of spatial and temporal variations in biofilm structure and activity. By elucidating the intricate interactions between biofilm organisms and their environment, these devices empower scientists to identify potential targets for biofilm control strategies and optimize the design of biofilm-based technologies for various applications. Ultimately, investing in devices to understand biofilm behavior not only enhances our fundamental knowledge of microbial ecology but also opens avenues for innovative solutions to address biofilm-related challenges in healthcare, industry, and environmental management (Wi and Patel, 2018).

## 3 Methods for studying biofilms

Biofilm studying methods aim to mimic natural conditions where biofilm grows, but in controlled laboratory settings. These methods could be classified into static and dynamic methods, based on the nutrients supply whether it’s continuous (dynamic methods) or limited to the initial conditions (static methods). The selected method of study determines the information that could be extracted from the experiment; therefore, choosing an appropriate biofilm studying method is vital (Alcàcer-Almansa et al., 2023).

### 3.1 Static methods

Static methods are characterized by their limited nutrient supply (Alcàcer-Almansa et al., 2023). Such methods include microtiter plates, colony forming units (CFUs), Calgary biofilm device (CBD) (Ceri et al., 1999), bio printed scaffolds (Ning et al., 2019; Aliyazdi et al., 2023), multielectrode arrays (Goikoetxea et al., 2018) as well as novel *ex vivo* models (Harrington et al., 2020; Konduri et al., 2021). Researchers commonly choose these methods when investigating microbial biofilms due to their advantages which include simplicity, high-throughput analysis and the ability to perform multiple tests under different conditions simultaneously. However, these methods exhibit specific limitations. First, they do not offer real-time information; instead, they provide only endpoint insights. Second, some of these methods involve washing steps, which can lead to an underestimation of the biomass. Lastly, integrating these methods with other techniques, such as microscopic techniques or electrochemical sensors can be challenging (Alcàcer-Almansa et al., 2023). Furthermore, it is important to note that the growing biofilms in these systems do not experience shear stress, and planktonic cells can be found in abundance in these systems. Consequently, these limitations combined can hinder the accuracy and repeatability of the experiments.

### 3.2 Dynamic methods

Dynamic biofilm methods involve a continuous flow system that supplies nutrients to the microbial biofilm. In these systems, a laminar flow passes over the growing biofilm, delivering necessary nutrients, removing planktonic cells, and eliminating metabolic waste. However, these methods tend to be more complex, expensive, and offer fewer high-throughput screening options compared to static systems. However, dynamic biofilm studying methods replicate *in vivo* conditions more closely compared to static systems. As a result, the outcomes produced by these methods are more reliable and better reflect the behavior of biofilms under natural conditions (Azeredo et al., 2017).

Flow chambers are the most commonly used dynamic methods to investigate microbial biofilms (Alcàcer-Almansa et al., 2023). This method relies on precise peristaltic pumps to continuously deliver culture media to a chamber where the biofilm forms. The chambers are enclosed with microscope cover slips. Within these chambers, the flow persists until the media is emptied into a waste container after passing over the biofilm surface. Moreover, these systems are versatile and can be customized for specific experimental setups (McGlennen et al., 2023a; Reichardt et al., 2024). Other examples of dynamic methods include Robbins device and rotary biofilm reactors (Han and Lee, 2023).

### 3.3 Microfluidic systems

Microfluidic devices represent a specific form of miniaturized dynamic biofilm studying methods, where fluids operate within microscale channels (Alcàcer-Almansa et al., 2023). These devices deliver a controlled laminar flow into the microchannels, over the biofilm to deliver required nutrients and wash away wastes and planktonic cells. Additionally, they offer many advantages including reduced reagent volume size, compact sizes, affordability and ease of operation (Alcàcer-Almansa et al., 2023; Yuan et al., 2023).

## 4 Microfluidic systems for biofilm study

Microfluidics refer to a range of technologies and applications that involve manipulating and controlling liquids at a micrometer scale (Yuan et al., 2023). These technologies offer multiple advantages in studying biofilms, and they can provide favorable conditions for biofilms growth and monitoring (Kim et al., 2012). Microfluidic devices enable high-throughput, real-time monitoring of biofilm dynamics under various conditions, allowing for biofilm investigation *in vitro* (Kim et al., 2012). Moreover, they could closely replicate physiological conditions, enhancing the reliability of outcomes in assessing antibiofilm compounds and investigating interactions within microbial biofilms (Terry and Neethirajan, 2014; Gomes and Mergulhão, 2021; Guzmán-Soto et al., 2021). Additionally, microfluidic systems offer a precise control over environmental parameters such as nutrient gradient (Tang et al., 2020), pH levels, and flow shear forces (Kim et al., 2018), which can be used to mimic the complex microenvironments where biofilms naturally grow. This level of control over such parameters allows researchers to investigate their effect on biofilm formation, structure and behavior with accuracy (Straub et al., 2020). Furthermore, microfluidic systems require minimal sample and reagent volumes, which makes using such systems favorable for researchers to reduce experimental costs and waste generation (Enders et al., 2022).

Microfluidic systems enable cultivation of different organisms in a microscale controlled environment, they offer many advantages such as rapid, real-time, low cost, high throughput bioanalysis (Zhang et al., 2019). This positions microfluidic systems as a promising avenue for biofilm study and monitoring. For instant, real-time analysis is a crucial advantage when studying biofilm behavior in order to extract useful information about the biofilm behavior at a microscale under different conditions (Straub et al., 2020). Moreover, the combination of microfluidic systems with other analytical techniques, such as microscopy and biosensors technology can provide a comprehensive and more versatile biofilm analysis (Liu et al., 2019; Blanco-Cabra et al., 2021). This can ultimately enable multidimensional insights into biofilm dynamics and behavior under specific conditions.

### 4.1 Microfluidic system design for biofilm study

Successfully designing a functional microfluidic system tailored to studying biofilm dynamics and behavior is a crucial step to ensure reliable and accurate experimental findings. Therefore, many parameters need to be considered and controlled in the designing process of the microfluidic system for biofilm study. These include microchannel dimensions (Blanco-Cabra et al., 2021), flow rate (Liu et al., 2019), surface properties, cells adhesion (Liu et al., 2019) and sensors integration into the microfluidic systems (Lederer et al., 2012). Additionally, choosing appropriate reagents, materials and microfluidic chip fabrication methods is essential to ensure biocompatibility and reduce the possibility of interfering with experimental results. By carefully considering these designing parameters, researchers can develop reliable microfluidic systems that can replicate *in-vivo* conditions, allowing for accurate reproducible biofilm research.

### 4.2 Microfluidic chip design and fabrication

A microfluidic chip is a miniaturized device which contains a network of channels that allow for fluid manipulation on a microscale, these channels can be designed to perform a variety of functions such as mixing, separation or carrying out bioreactions (Pattanayak et al., 2021). In a recent review, Pérez-Rodríguez et al. have ingeniously categorized the numerous microfluidic chips with different designs into six distinct groups: (i) devices with linear channels, subdividing for one, two and three parallel channels; (ii) devices with mixing channels; (iii) devices with multiple floors; (iv) porous devices; (v) topographic devices and (vi) droplet microfluidics (Pérez-Rodríguez et al., 2022). In biofilm research context, microfluidic channels are designed to facilitate biofilm adhesion, formation, and growth under dynamic controlled conditions. Therefore, the design of the channels plays a significant role in governing the dynamics of biofilm growth and formation. Consequently, the geometric features and surface properties of these channels present a pivotal influence on the biofilm adhesion and formation. For instance, Blanco-Cabra et al. investigated different channel geometries and their effect on biofilm formation, where they have optimized the channels geometry and dimensions to enable optimal biofilm formation (Blanco-Cabra et al., 2021). Additionally, it was highlighted that the channels geometry and shear stress impacted the growth of biofilm, which algins with a similar previously documented study (Salek et al., 2009). Moreover, microfluidic chips can also offer the versatility of integrating biosensors, which allows to create a platform for studying biofilm dynamics.

Various substances may be used to create microfluidic chips, and each has certain benefits and characteristics of its own. Thus, choosing the best material for device manufacturing is one of the essential stages in microfluidic applications (Niculescu et al., 2024). There are various examples of materials that are commonly used in the fabrication process include glass, silicon, and polymers, each offers its own advantages and disadvantages depending on the specific intended application (Niculescu et al., 2021). Microfluidic chips have witnessed material transitions over time. Initially crafted from silicon (in the 1960s) or glass (in the 1970s), polymer-based materials were introduced in the 1990s, followed by paper-based microchips in the 2000s. The adaptation and widespread use of microfluidic chips has been driven by their manufacturing and handling characteristics. When fabricating microfluidic devices for biofilm studies, some important properties need to be considered. These include material biocompatibility (Elvira et al., 2022), optical transparency for microscopic applications (Gharib et al., 2022), the ability to fabricate micro-scale channels and the capacity for surface functionalization to promote proper cell adhesion (Ren et al., 2013). These considerations are important to achieve accurate and reliable results.

The choice of fabrication technique depends on many factors including the selected materials compatibility, cost and scalability (Niculescu et al., 2021). Microfluidic chips can be fabricated using various techniques (Niculescu et al., 2021), and they have been classified in literature based on how the microfluidic structures are created (Waldbaur et al., 2011), either by removing or depositing material. Another classification breaks down the fabrication techniques into categories based on the types of processes that are used, such as chemical, mechanical, laser-based, and other processes (Hwang et al., 2019). Examples with classifications were presented by Niculescu et el (Niculescu et al., 2021).

Silicon microfluidic chips are fabricated using three essential processes: photolithography, etching, and doping. These chips exhibit remarkable precision, but their inherent opacity poses challenges for microscopic research using transmitted or reflected light. Consequently, direct observation of microfluidic chips using traditional optical microscopy techniques becomes difficult. However, glass-based microchips provide a simpler and more user-friendly alternative. Their fabrication process involves laser cutting individual glass layers and thermo-compression bonding multiple layers. Despite its fabrication simplicity, challenges arise during the bonding of multiple glass layers due to the need for precise alignment and sufficient bonding between the glass plates. The complexity, manual operation, and low productivity associated with the bonding step further exacerbate these challenges (Han et al., 2021).

The fabrication of both glass and silicon microfluidic chips involves complex, costly and time-consuming processes. The microfluidic chips produced are fragile and the cost of producing them is high. Consequently, this makes silicon and glass microchips less attractive than the next generation polymer-based multilayer chips, such as polymethyl methacrylate (PMMA) or polydimethylsiloxane (PDMS). Those polymers are relatively inexpensive compared to materials such as silicon and glass and the fabrication is easy to process (Niculescu et al., 2021).

PMMA is a transparent thermoplastic polymer synthesized from the monomer methyl methacrylate. PMMA can be a cost-effective replacement for glass. The fabrication of multi-layer PMMA chips employs laser cutting (Chen et al., 2019) and plasma-activated thermal bonding (Trinh et al., 2020). PMMA offers excellent mechanical stability and rigidity. Its transparency in the visible light spectrum makes it suitable for applications involving optical detection methods, such as fluorescence microscopy and absorbance spectroscopy. However, some solvents or reagents can interact with PMMA, altering its properties and causing leaching of monomers. Additionally, it is relatively sensitive to scratches and can be brittle, especially in thin structures.

Another replacement for glass in the microfluidic chips is cyclic olefin copolymer (COC), a relatively new polymer, composed of cyclic olefin monomers (norbornene) and linear olefins (ethylene) (Shin et al., 2005). Its advantages include good biocompatibility, low water absorption properties, heat-resistant, good chemical resistance and high transparency in the deep UV range, minimizes interference in fluorescence-based assays (Agha et al., 2022). However, the price is relatively higher than PMMA or PDMS.

The above materials used in the manufacture of microchips are inferior in popularity to polydimethylsiloxane, commonly referred to as PDMS or dimethicone. PDMS is a mineral-organic polymer composed of carbon and silicon. It belongs to the siloxane family and is achieved by mixing elastomer (base polymer) and curing agent (a cross-linker) and adjusting thermosetting characteristics (curing time, temperature, degree of cross-linking and final hardness and strength) (Torino et al., 2018). Following cross-linking, PDMS transforms into a hydrophobic elastomer. However, the hydrophilic properties of a material are crucial in microfluidic chips for several reasons, including fluid flow control, reducing air bubbles formation, enhanced cell adhesion, and minimized protein adsorption. Therefore, the surface chemistry of PDMS can be modified through oxidation by radiofrequency or oxygen plasma treatment resulting in the introduction of silanol terminations (Sih). This could also be achieved by using functional organic compounds such as 2-hydroxyethyl methacrylate (HEMA), polyethylene glycol (PEG) and polyvinylpyrrolidone (PVP). Consequently, the material temporarily becomes hydrophilic (Hemmilä et al., 2012) and ready to be used.

PDMS is exceptionally soft and deformable. Its elasticity allows it to undergo significant deformations without damage and making it suitable to integrate inlets and outlets including valves and pumps, directly into the microfluidic chips structure (Rahimnejad et al., 2022). Unlike rigid materials like glass and silicon, PDMS can bend, stretch, and conform to various shapes. PDMS can be molded, cast, and patterned easily (Lin and Chung, 2021). Beside the ease of fabrication, PDMS possesses the features of optical transparency and gas permeability which can be advantageous for oxygen exchange in cell culture applications as well as non-toxicity and biocompatibility, making it suitable for long-term cell culture, cell screening, and biochemical studies (Auner et al., 2019).

Although PDMS is considered the golden standard for microfluidic device materials, it does have certain drawbacks. For instance, during PDMS curing, some residual polymer chains remain incompletely crosslinked, and these oligomers can diffuse freely within the PDMS material and may leach out when exposed to a solution (Regehr et al., 2009; Wang et al., 2018). Additionally, PDMS can absorb small molecules due to its porous nature, this includes molecules found in cell culture media as well as test substances. Consequently, this makes PDMS based microfluidic chips less suitable for pharmaceutical experimentation (Toepke and Beebe, 2006; Su et al., 2011; Wang et al., 2018). Moreover, organic solvents could also be absorbed by PDMS’s porous structure leading to deformation of microchannels (Raj and Chakraborty, 2020). Furthermore, high flow rates can also induce the expansion and deformation of PDMS microchannels (Jia et al., 2022). To address the challenges associated with PDMS, researchers use various strategies. One effective approach involves surface modification of microchannels using non-stick and inert materials such as sol-gel-based silica nanoparticles, Parylene, and perfluorinated polymers like Teflon AF and Cytop (Gomez-Sjoberg et al., 2010; Sasaki et al., 2010; Carlborg et al., 2011; Teng, 2012; Wang et al., 2018). Another alternative to PDMS is off-stoichiometry thiol-ene (OSTE). In the fabrication of an OSTE-based microfluidic chip, PDMS serves as an intermediate mold for injection and curing. However, the final OSTE-based microfluidic chip does not incorporate PDMS, as OSTE effectively confines the channels and chambers (Priedols et al., 2023; Rimsa et al., 2021).

Selecting proper materials and fabricating techniques are crucial steps in the process of designing a microfluidic chip that is able to mimic biological conditions and offer a versatile platform to study biofilm behavior under controlled conditions. Therefore, researchers should carefully consider factors such as biocompatibility, chemical resistance, optical transparency, and ease of fabrication when designing microfluidic chips.

### 4.3 Biosensor integration with microfluidic systems

The integration of electrochemical biosensors into microfluidic systems offers a synergistic platform that significantly advances the study of biofilms (Yawata et al., 2016). Microfluidic systems are known for their ability to monitor events as they happen, and they easily align with the real-time capabilities of electrochemical biosensors. The synergy allows for clearer real-time insights into biofilm behavior, while providing a controlled microenvironment. This could additionally allow for enhanced sensitivity and precision of the electrochemical biosensor (Fernández-la-Villa et al., 2019; Song et al., 2019). These enhanced capabilities provide opportunities for research into the dynamics of biofilms, the effects of antimicrobial agents, and the microbial response to alterations in the microenvironment.

## 5 Electrochemical biosensors

Electrochemical biosensors are analytical devices that combine biological recognition elements with electrochemical transducers to detect and quantify target analytes by generating electrical signals (Singh et al., 2021). Furthermore, due to their non-destructive and non-invasive nature, it is regarded as a direct *in-situ* detection and monitoring method in living systems (Suhito et al., 2020). Electrochemical biosensors present many advantages including their ease of use, real-time highly sensitive and specific responses (Saulnier et al., 2024). Additionally, they present a high potential for miniaturization and incorporation into microscale systems (Zhang et al., 2020). This results in reducing the dependence on chemical agents such as dyes, fluorogenic and molecular probes. These characteristics position electrochemical biosensors as a promising approach for studying microbial biofilm dynamics and response to various antibiofilm agents.

**Figure 3:**
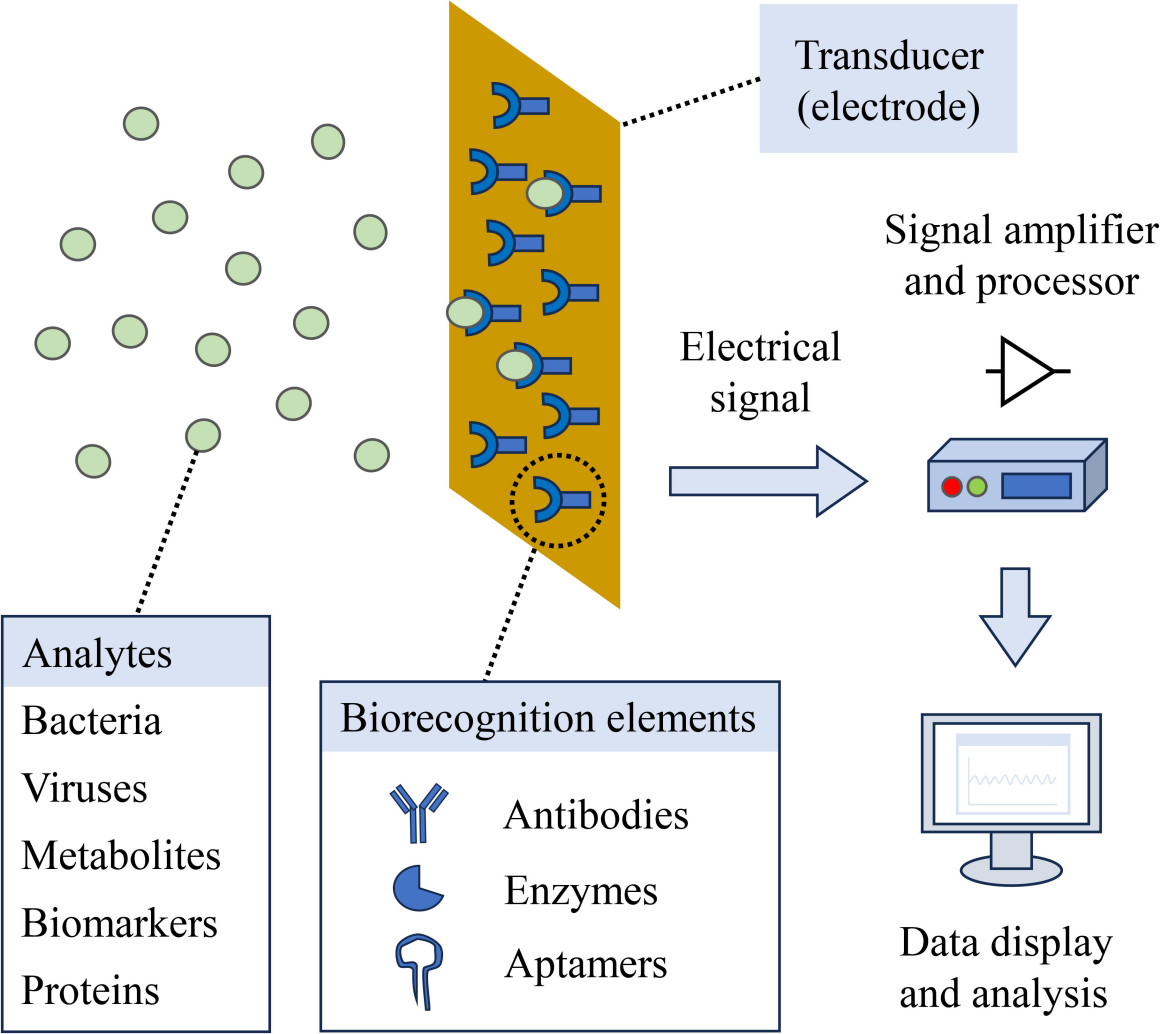
Electrochemical biosensor main components.

Electrochemical biosensors consist of a biorecognition element, transducer, signal amplifier, processor, and a display or suitable output (Grieshaber et al., 2008; Shanbhag et al., 2023) (See **Figure 3**). These constitute a versatile platform for detecting biofilms and their metabolic activities. These systems convert biochemical interactions into detectable electrical signals, operating through diverse methods of analysis like amperometry, potentiometry, or impedance (Shanbhag et al., 2023). Many papers have reported using diverse electrochemical methods for real-time studying and monitoring of biofilms activity (Becerro et al., 2016; Liu et al., 2018; Gupta et al., 2019; Andriukonis et al., 2021; Liu et al., 2021). For these methods to be efficient and reliable in studying biofilm behavior, they should fundamentally possess these analytical parameters (**Table 1**) to evaluate their performance and output (Stoytcheva et al., 2009; Shanbhag et al., 2023).

**Table 1:** Key analytical parameters for reliable electrochemical results for biofilm behavior monitoring (Shanbhag et al., 2023).

Analytical parameter	Definition
Sensitivity	The electrochemical biosensor must be sensitive to changes in analytes’ concentration (ng/mL up to fg/mL). To be able to detect changes in biofilms behavior under different conditions.
Selectivity	The response of the electrochemical biosensor must be selective to the desired analytes, with minimum or no cross-reactivity. To prevent false interpretation of the electrical responses.
Response time	To promote real time *in-situ* study of biofilms dynamics, a short response time of the electrochemical biosensor must be achieved.
Reproducibility	Same analytical signals must be generated with the same magnitude when repeating the measurements.
Stability and lifetime	The measured responses need to be dynamic and stable throughout the measurements to provide a long-term monitoring of the biofilm behavior under various changes in conditions such as media, temperature or pH.

Despite the growing number of reports from research groups on novel electrochemical biosensor development, many challenges need to be fully addressed (Ferrag and Kerman, 2020). These challenges include achieving a low limit of detection (LOD) and ensuring repeatability and reproducibility which are crucial for reliable interpretation of the data to understand biofilm dynamics. Additionally, it is important to maintain a reproducible and stable environment for the biosensor to operate effectively, in some cases for up to 72 hours of continuous monitoring (Liu et al., 2021). These challenges have been addressed by using different approaches and methodologies. This includes nanoparticles functionalization (Pingarrón et al., 2008; Ferrag and Kerman, 2020; Cai et al., 2023), and biosensor incorporation into microfluidics systems, which could increase sensitivity, specificity and reduction in reagent consumption (Chircov et al., 2020).

## 6 Electrochemical techniques

The investigation of microbial biofilms requires modern techniques that can provide accurate and real-time insights of the biofilm dynamics. Electrochemical techniques have emerged as invaluable means to explore biofilms and their responses to different stimuli and conditions. As electrochemical sensing is a non-destructive method, the biofilm remains intact in most cases and can continue to grow and develop after the measurements. This is important for monitoring biofilms continuously and collect data at any important stage, as it does not disrupt the biofilm structure. Also, electrochemical sensing can be used to monitor a wide range of parameters related to biofilm growth and activity, including metabolic activity, cell density, and changes in pH or other environmental factors. Such advantage makes electrochemical sensing a versatile tool for studying biofilms in different settings and under different conditions. Electrochemical sensors are known for their short response time and simple fabrication. Finally, electrochemical sensors can be effective in terms of costs, especially with the potential for miniaturizing (Pousti et al., 2019; Subramanian et al., 2020; Magar et al., 2021).

### 6.1 Electrochemical impedance spectroscopy

Electrochemical impedance spectroscopy (EIS) is a powerful technique used to characterize and understand the liquid-solid interface. Obtaining an EIS spectra involves applying a small sinusoidal potential (or current) and recording the current (or potential) whilst sweeping slowly across a range of frequencies, with each input signal equally spaced on a descending logarithmic scale from ~10 kHz - 1 MHz to a lower limit of ~10 mHz - 1 Hz. EIS does not involve redox reactions; thus, the method can be used over time without a risk to damage the biofilm structure. When the biofilm is exposed to an electric field, the biofilm cells act as an insulating layer that impede the flow of electric signal, thus increasing the impedance of the system.

Electrochemical impedance spectroscopy in microfluidic chips is a powerful technique for studying microbial biofilms. In microfluidic setups, EIS helps analyze microbial biofilms’ electrical properties, providing insights into their structure, growth and behavior (Pousti et al., 2019; Magar et al., 2021). This technique aids in understanding how microorganisms interact within confined spaces, contributing to advancements in microbial biofilm research and applications in areas like biofilm-based sensors and drug delivery systems. For instance, in (McGlennen et al., 2023a) a novel highly stable 3D printed flow cell system to analyze biofilms was developed with microfabricated EIS sensors combined with live-cell confocal laser scanning microscopy imaging to thoroughly interpret EIS data from biofilms over various stages of growth. The system has potential to analyze biofilms of different species. In a different study EIS biosensors in microfluidic system were employed to monitor various biofilm treatment responses using anti-biofilm agents. The microfabricated biosensors were effective at assessing the growth and removal of *P. aeruginosa* biofilm. The system was effective in metalworking fluid, which allows evaluation in industrial settings (McGlennen et al., 2023b).

The EIS-based methods for biofilm detection are more amenable to miniaturization compared to faradaic methods. As EIS does not involve redox reactions, there is no need for a reference electrode. However, to interpretate EIS data, certain knowledge of electrochemistry and biofilm physics is required and may be complicated for inexperienced researchers. EIS experiments are time consuming as multiple measurements at different frequencies are required.

### 6.2 Voltammetry

Microbial metabolism entails redox reactions to sustain the energy demand of the biofilm. Extracellular electron transfer permits the exchange of electrons between the cells in the biofilm and surfaces they are attached on, which drives biochemical reactions necessary to convert substrates to products. The electrode-reducing extracellular electron transfer describes electron flow from the cell to the surface. On the contrary, electrode-oxidizing extracellular electron transfer relates the flow of electrons from surface to cell. Microbes transfer electrons either directly to redox-active surfaces or indirectly via conductive mediators (e.g. soluble electron shuttles). Two commonly found and well-described electroactive species belong to *Geobacter* and *Shewanella* spp. They use the anode electrode as the terminal electron acceptor and are commonly referred as exoelectrogens. The passage of the electrons occurs via electrically conductive protein nanowires, referred to as e-pili, and cytochrome c proteins. The electrotrophic microbes can take up electrons from solid-state donors such as the cathode for the reduction of terminal electron acceptors (Kang et al., 2012). The electroactivity of the biofilm is essential feature to analyze it electrochemically applying techniques where Faradaic processes are involved. Voltammetry encompasses a group of electrochemical techniques where interfacial electron or ion transfer is induced by externally applying potential difference in an electrolytic cell (Harnisch and Freguia, 2012; Kang et al., 2012). In the following subsections several voltametric techniques applied in biofilm analysis are briefly described.

#### 6.2.1 Cyclic voltammetry

Cyclic voltammetry (CV) is a technique applied to record the current response of an electrically active species to a linearly cycled potential sweep, cycling from an initial to final polarization. The scan rate of the forward and backward voltage sweeps can be varied from 1 to 100mV/s. In a CV plot, anodic and cathodic peaks are registered if a redox system is reversible; while for an irreversible system, only a single peak is observed. As the precision of redox peak separation is influenced by the scan rate, it should be chosen carefully based on the objective of the experiment. It is an informative method if one needs to analyze the thermodynamics of redox processes, the energy levels of the analyte and the kinetics of electronic-transfer reactions of the system. Cyclic voltammetry is the tool to distinguish between surface controlled and diffusion‐ controlled processes. In case of surface‐controlled processes, the reaction occurs at the electrode surface. The peak intensity in voltammogram is directly proportional to the scan rate. On the contrary, if the process is diffusion‐controlled, the redox species is a part of bulk electrolyte instead of being attached to electrode surface, and its oxidation–reduction would be dependent on its rate of diffusion from the bulk toward the electrode. Peak intensity in diffusion‐ controlled processes is proportional to square root of the scan rate.

Electron transfer processes in a biofilm can be studied by CV as it differs depending on development stages and biofilms communities. Mechanisms of electron transfer, whether due to unrestricted circulating species or surface‐adsorbed species by the microbial biofilms, can be detected with the help of CV (Pousti et al., 2019).

The detection of biofilms by cyclic voltammetry was demonstrated by the changes in the cyclic voltammograms, namely the increase of the current indicating the increased electron transfer at the beginning of the biofilm formation and the decreases in the anodic and cathodic currents at the later biofilm development stages. Microbial adsorption and biofilm formation reduce the surface area of Au electrode thus decreasing the current in cyclic voltammogram. The method was validated with microscopic image of the electrode surface at different biofilm development stages (Kang et al., 2012).

#### 6.2.2 Square wave voltammetry

In a square wave voltammetry (SWV) experiment, the working electrode’s potential is stepped through a sequence of forward and reverse pulses, starting from an initial potential and reaching a final potential. The forward step is set by the square amplitude, while the reverse step is calculated by subtracting the square increment from the square amplitude. This technique is employed for registering of low concentration redox species secreted by biofilms. Besides, square-wave voltammetry can aid in predicting the reversibility of an electrochemical reaction by assessing the ratio of peak currents’ magnitudes between the forward and reverse scans.

The successful employment of the method is demonstrated in several works with electrodes on microfluidic chips. The presence and concentration with linear response from 0.1 μM to 10 μM of flavins inside a *S. oneidensis* MR-1 biofilm was detected with a microelectrode using SWV (Nguyen et al., 2012). Precise electrochemical measurements of pyocyanin SWV are established using carbon fiber assemblies. The electrode allowed linear quantification from 1 µM to 100 µM of pyocyanin (Sharp et al., 2010).

#### 6.2.3 Differential pulse voltammetry

Differential pulse voltammetry (DPV) involves scanning potential by applying a series of pulses to the electrode, each kept at a small, fixed amplitude. The current is monitored at the beginning and end of each pulse and plotted to create a peak-shaped voltammogram. DPV is favored due to its differential approach, reducing the impact of background charging current during analysis. Its short pulses contribute to high sensitivity, enhancing accuracy. This technique is valuable for both qualitative and quantitative analysis of redox species (Kumar et al., 2019). The biofilm of *G. uraniireducens* was characterized employing DPV. The redox peak current at -176 mV increased with time and was attributed with riboflavin (Huang et al., 2018).

#### 6.2.4 Chronoamperometry

Chronoamperometry (CA) is a voltametric technique where the applied potential is fixed through all the experiment during which faradaic current is measured with respect to time. CA has various applications, including identifying electrogenic species within biofilms, forming electroactive biofilms, and distinguishing between capacitive and faradic currents. Moreover, CA can evaluate the operational status of a biofilm, as the produced bioelectricity correlates with cell growth. Consequently, CA experiments can roughly estimate the lag, log, stationary, and decline phases of microbial culture based on current output. When integrated with other electrochemical techniques, CA offers a broad characterization profile for the biofilm (Sevda et al., 2018; Kumar et al., 2019; Funari and Shen, 2022).

The characterization of biofilm formation of *G. sulfurreducens* on indium-tin oxide (ITO) electrode employing CA was successfully performed. A rapidly increasing current as a result of catalytic oxidation of acetate in the *G. sulfurreducens* biofilm growing at the ITO electrode was observed (Jain et al., 2011).

### 6.3 Potentiometric measurements

In potentiometric analysis, a reference electrode and an indicator electrode are placed in a basic electrochemical cell. The potential difference between these electrodes is measured to yield information about the analyte concentration. In this setup, there is either no current flow or only a negligible amount, emphasizing the sensitivity of the technique (Kumar et al., 2019; Funari and Shen, 2022).

Biofilms can be considered as a battery or capacitor, where the transmembrane potential is defined by their metabolic state. It only takes the transfer of ~100 monovalent ions to change a bacterial membrane potential by a millivolt assuming a cellular capacitance of 1 μF/cm^2^. The data obtained from biofilm-coated electrodes indicate that there is a positive linear correlation between capacitance and the number of living cells within a biofilm. And vice versa, there is a negative linear correlation observed for the open circuit potential with respect to the number of living cells (Saboe et al., 2021).

## 7 Applications in studying biofilm behavior

**Figure 4:**
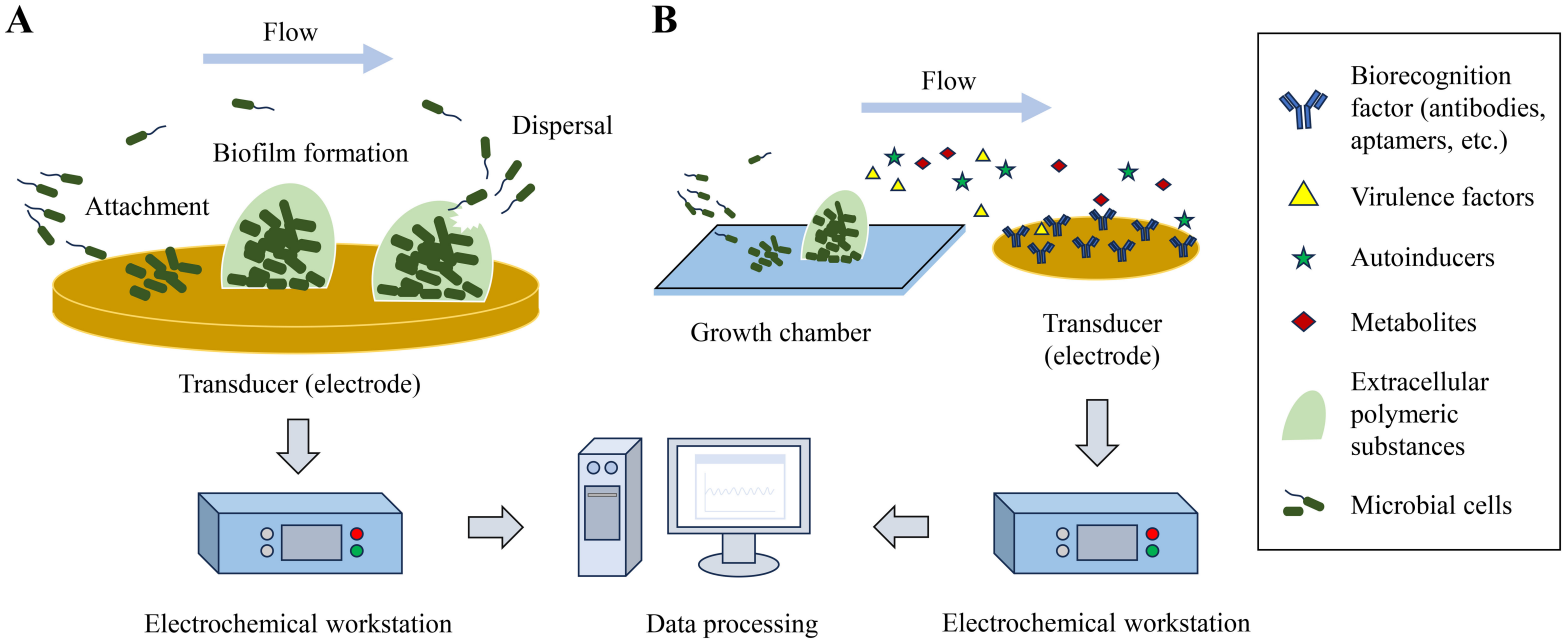
Schematic of electrochemical biosensor for biofilm study under flow. **(A)** Biofilm formation and dispersal on an electrode and being monitored by an electrochemical workstation. **(B)** Detection of biofilm metabolites using a growth chamber and a modified transducer connected to an electrochemical workstation.

Microbial biofilms are dynamic and complex communities of bacteria, that communicate with each other to ensure higher chances of survival (Hall-Stoodley et al., 2004). Thus, understanding their behavior in a dynamic setting is crucial for developing countermeasures. The integration of electrochemical biosensors with microfluidic platforms offers a versatile and dynamic controlled environment for assessment of different microbial biofilm aspects (**Figure 4**). This synergy allows for a more comprehensive understanding of microbial biofilms dynamics to develop more efficient interventions in various fields. Therefore, researchers have applied these microfluidic devices facilitating diverse systems for various applications, as shown in **Table 2**.

**Table 2:** Microfluidic device data for the electrochemical technique used, target microorganism, purpose of the study, device setup, experiment duration, and whether it is possible to test antibiofilm agents using the device.

Ref.	Electrochemical technique used	Target Microorganism	Purpose of the study	Microfluidic device setup	Exp. duration	tested antibiofilm agents
(Pires et al., 2013)	Electrochemical Impedance Spectroscopy (EIS)	*P. aeruginosa*	To provide decisive information about biofilm growth dynamics and effects of different treatment strategies.	The system consists of two microfluidic channels, reference and measurement channel. Each channel is equipped with four sets of planar electrodes to perform EIS measurements.	72 h	Yes
(Song et al., 2023)	Electrochemical Impedance Spectroscopy (EIS)	*Streptococcus mutans* (*S. mutans*)	To perform automated and continuous anti-biofilm drugs screening and biofilm monitoring.	Microfluidic chip was assembled to form a closed micro-reaction tank with G-MEAs placed at the center. The chip is connected to a peri-static pump and a waste pool on both sides of the chip as an inlet and outlet, respectively.	72 h	Yes
(Blanco-Cabra et al., 2021)	Electrochemical Impedance Spectroscopy (EIS)	*Pseudomonas aeruginosa* PAO1. *Staphylococcus aureus* (ATCC12600)	To investigate polymicrobial biofilms susceptibility to antibiotics, in a dynamic simulating environment.	The chip includes several sets of independent chambers, each connected to two inlets, one chamber and three different growth chambers. The growth chamber is integrated with gold sensor to perform EIS measurements.	Growth phase 60-80 hours and a treatment phase 16-24 hours.	Yes
(Subramanian et al., 2017)	Impedance	*E. Coli*	To perform real-time biofilm detection and treatment with a threshold-activated feedback-based impedance sensor-treatment system.	The microfluidic chip is integrated with interdigitated gold microelectrodes and connected to a Potentiostat to perform impedance measurements. to allow dynamic flow, the chip was fabricated to have inlets to introduce media and outlet for waste.	24 h	Yes
(Claydon et al., 2016)	Electrochemical Impedance Spectroscopy (EIS)	*P. aeruginosa*	To perform a non-destructive biofilm characterization in a dynamic flow system.	The microfluidic flow cell was developed to create a sheath flow in the central measurement chamber. The chamber was equipped with working electrodes to perform EIS measurements.	–	No
(Pitruzzello et al., 2023)	Electrochemical Impedance Spectroscopy (EIS)	*E. coli*	To identify specific bacteria and track the change of their metabolic response to an antibiotic exposure.	Glass microscope slides with built-in microelectrodes and PDMS channels made up the microfluidic system. The glass slide was adhered to by the PDMS device, which has microfluidic channels, to provide a substrate for regulated fluid flow. The investigation of biofilm behavior on integrated microelectrodes was made easier by this approach.	–	Yes
(Liu et al., 2021)	differential pulse voltammetry (DPV)	*P. aeruginosa*	To monitor the biofilm growth and its relation to virulence factor production (pyocyanin)	The microfluidic chip is designed to have multiple growth chambered which are integrated with microelectrodes and connected to an electrochemical workstation to perform DPV measurements. It is equipped with inlets and outlets to maintain a dynamic microenvironment.	72 h	Yes
(McGlennen et al., 2023b)	Electrochemical Impedance Spectroscopy (EIS)	*P. aeruginosa*	To perform early detection and continuous monitoring of biofilms. Additionally, to test QS effect on biofilm growth rate.	A 3D printed flow chip system integrated with electrochemical biosensor to perform EIS measurements. The flow chip was equipped with inlet and exit ports to maintain a dynamic controlled environment.	36 h	Yes
(Liu et al., 2018)	Electrochemical Impedance Spectroscopy (EIS)	*Salmonella.* *E. coli.*	To monitor the biofilms formation process and development in real-time using impedance detection.	The microfluidic chip containing growth micro cavities was integrated with gold electrodes. The electrodes were connected to electrochemical workstation to perform EIS measurements. The microfluidic chip was equipped with outlets and inlets to insure a dynamic flow of media.	48 h	Yes
(Bruchmann et al., 2015)	A combination of amperometry activity measurement and EIS	*P. aeruginosa and S. maltophilia*	To determine biofilms viability and to estimate the attached biofilm biomass	A microfluidic system equipped with 12 fluidically independent microfluidic channels, in parallel. The chip is integrated with electrodes to perform electrochemical measurements.	72 h	Yes

### 7.1 Monitoring biofilm proliferation

The real-time capabilities of electrochemical biosensor integrated microfluidic platforms provides a valuable insight into the growth and proliferation dynamics of biofilms. For instance, Blanco-Cabra et al. employed this approach in designing a microfluidic integrated chip to monitor the growth and treatment of different strains of *P. aeruginosa* and *S. aureus* biofilms in a controlled manner. The chip is composed of three independent growth chambers integrated with electrodes to perform impedance measurements under a controlled flow rate. This system allows for a homogenous biofilm formation and growth while mimicking physical conditions encountered in natural environments. By integrating electrical impedance spectroscopy, the microfluidic device can be utilized to investigate novel anti-biofilm tactics (Blanco-Cabra et al., 2021). This system has been designed and tested to grow biofilms with diverse characteristics, including bacterial species from laboratory use or clinical use. To further assess its applicability in susceptibility testing, sputum samples from patients with lung infections were used to further study the usefulness of susceptibility testing, which helps in determining the most effective therapy for a customized medication (Blanco-Cabra et al., 2021). Similarly, Subramanian et al. demonstrated the use of a microfluidic system with integrated microelectrodes to perform impedimetric measurements to accurately monitor bacterial biofilm growth and proliferation. This innovative study introduced a novel threshold-activated feedback-based impedance approach to real-time monitoring and treatment of biofilm growth (Subramanian et al., 2017). The integration of electrodes into microsystems presents a significant opportunity for novel and promising approaches to precisely monitor the growth of biofilms in real time and to screen for novel therapeutic strategies (McGlennen et al., 2023b).

### 7.2 Microbial communication and virulence dynamics

Quorum sensing molecules mediate biofilm communication, playing a pivotal role in regulating gene expression, virulence factors, antibiotic resistance, and proliferation (Sharma et al., 2019). Therefore, their detection and quantification are key steps in understanding the intricate biofilm dynamics. Electrochemical biosensor integrated on microfluidic chips provide a reliable real-time platform to monitor the presence and concentration of quorum sensing (QS) molecules. Moreover, these platforms provide crucial insights into the concurrent adjustment of both virulence factors and biofilm communication. Their potential is significant in combating biofilm-related infections, offering applications like point-of-care diagnosis and dynamic platforms for screening potential anti-biofilm agents.

Pyocyanin is an example of a specific virulence factor and QS signaling molecule in Pseudomonas aeruginosa, its redox-active nature makes it a well-suited candidate for electrochemical detection. In their work, Liu et al. engineered a microfluidic chip with integrated microelectrodes, providing a controlled environment for the analysis of biofilm growth and the secretion of pyocyanin (Liu et al., 2021). The microfluidic chip was equipped with growth chambers and integrated with microelectrodes connected to an electrochemical workstation to perform differential pulse voltammetry (DPV) for pyocyanin quantification. The study demonstrated a close correlation between pyocyanin concentration and the formation and growth process of the biofilm, with a rapid concentration increase during the biofilm growth phase followed by a decline during the biofilm dispersal stage. Additionally, they investigated the impact of curcumin on the production of pyocyanin, and it was revealed that the presence of curcumin notably decreased the pyocyanin concentration (Liu et al., 2021). Their design provided an *in-situ*, continuous, and non-invasive method of monitoring biofilm growth and pyocyanin secretion, which shows a great potential to be used in various applications in managing biofilm-related infections. The previous study highlights the potential of electrochemical biosensor-integrated microsystems to comprehensively investigate biofilms dynamics. This approach contributes to our understanding of bacterial biofilm communication and virulence.

### 7.3 Assessment of biofilm antimicrobial susceptibility

The continuous ongoing rise in bacterial resistance highlights the pressing demand for robust, high throughput techniques to evaluate the susceptibility of biofilms to antimicrobial treatments (Gajic et al., 2022). In addressing this challenge, antibiotic susceptibility testing (AST) plays a pivotal role, offering valuable insights into the efficacy of antibiotics against bacterial infections. Within this landscape, electrochemical biosensor integrated microfluidic systems emerged as transformative tools, offering high throughput platforms for rapid antimicrobial screening (Khan et al., 2019), their utilization in AST field brings multiple advantages such as reduced sample volume, real-time monitoring, cost effectiveness. For instance, the microsystems developed by Blanco-Cabra et al. showed a great potential for providing a straightforward tool for antimicrobial biofilm susceptibility testing on clinical samples, it provided a dynamic mimicking environment, while producing robust results. The transformative impact of electrochemical biosensor integrated microfluidic systems in antibiotic susceptibility testing is evident, providing a significant advancement in our ability to quickly and effectively evaluate biofilm susceptibility to antimicrobial treatments.

## 8 Conclusions and future perspective

As the threats of microbial biofilm infections grow, there is an increasing demand for a versatile and *in-situ* novel systems for biofilm research. While existing macroscale systems offer insights into biofilms, they often limit researchers to end-point characterizations that are invasive and destructive (Alcàcer-Almansa et al., 2023). Electrochemical biosensor integrated microfluidic systems offer a variable and innovative solutions to studying biofilm dynamics. Integrating electrochemical biosensors with microfluidic systems offers a synergetic platform that combines the advantages of both. This synergy creates a real-time, non-invasive method for studying biofilm behavior in a controlled microenvironment. These systems can serve multiple applications in biofilm studies including monitoring biofilm proliferation, communication, virulence, and antimicrobial susceptibility. Their efficiency in biofilm research extends to the development and testing of novel therapies, reducing reagents and resources costs. However, there are still challenges that need to be addressed such as sensitivity, selectivity, response time and reproducibility of these systems. Furthermore, microfluidic investigations of microbial biofilms pose additional challenges. Microchannel clogging can cause flow inconsistencies, while the risk of contamination during incubation and experimentation remains a significant concern due to the nutrient-rich media flowing through the microchannels (Yuan et al., 2023). Future research in this dynamic field has the potential to address these challenges and to elevate the capabilities of these integrated system.

To address these challenges researchers have attempted many designs and solutions. For instance, possible contamination can be avoided by using different measure such as using sterile filters for the tubing systems, thoroughly sterilizing the tools used in the system (Yuan et al., 2023). Additionally, channels clogging and biofouling could be minimized using various designs and techniques, for example, one design incorporates three inlet channels that converge into a single chamber, followed by an outlet channel. This flow-focusing arrangement allows precise control of flow rates from three distinct liquid reservoirs, enabling spatially separated flows of different media within the same chamber due to the laminar flow arrangement (Straub et al., 2020). In addition to that, biosensors and associated electrochemical measurements can yield substantial data volumes, which often pose challenges in interpretation due to their complexity and resource-intensive nature. Consequently, there is a high risk of misinterpreting the data and being overwhelmed by the volumes produced by the electrochemical measurements. Therefore, the development of deep learning algorithms can be crucial to analyze and handle the produced data accurately and effectively (Cui et al., 2020; Raji et al., 2022). Finally, a smart system has also been proposed to automate microfluidic integrated biosensors and ensure an accurate real-time detection of the onset of biofilms and their *in situ* treatment (Subramanian et al., 2017), which hold a great potential for future development of optimized algorithm-powered systems.

As we progress, it becomes evident that the current systems under development hold exciting potential for understanding the complex dynamics of biofilm behavior. Nevertheless, a crucial avenue for further research lies in optimizing these systems to enhance their efficacy. It is imperative to extend the scope of research by delving into the complexities of polymicrobial biofilms, unravelling different microbial species coexistence and interactions in biofilms. Additionally, incorporating real-world samples will be instrumental in bridging the gap between controlled laboratory conditions and practical applications. It is also essential to investigate a wider range of antibiofilm agents, including those that target QS pathways, to fully assess the effectiveness of these systems. Additionally, future work on developing organ-on-chip systems in conjunction with biofilm research stands as an exciting frontier. This can provide a simulated environment that closely resembles natural physiological settings that could provide valuable insights on how biofilms react to different conditions. Conclusively, the integration of electrochemical biosensors into microfluidic chips stands as a pivotal force propelling biofilm research forward.
